# Electromagnetic Exposure of Personnel Involved in Cardiac MRI Examinations in 1.5T, 3T and 7T Scanners

**DOI:** 10.3390/ijerph19010076

**Published:** 2021-12-22

**Authors:** Katarzyna Sklinda, Jolanta Karpowicz, Andrzej Stępniewski

**Affiliations:** 1 Department of Radiology, Centre of Postgraduate Medical Education, Marymoncka 99/103, 01-813 Warszawa, Poland; katarzyna.sklinda@gmail.com; 2 Department of Bioelectromagnetics, Central Institute for Labour Protection–National Research Institute (CIOP-PIB), Czerniakowska 16, 00-701 Warszawa, Poland; 3 ECOTECH-COMPLEX Centre, University of Maria Curie-Skłodowska, Głęboka 39, 20-612 Lublin, Poland; astep@ipan.lublin.pl

**Keywords:** static magnetic field, movement-related vertigo, workers’ safety, environmental engineering, biomedical engineering, radiology, COVID-19

## Abstract

(1) Background: It has been hypothesised that a significant increase in the use of cardiac magnetic resonance (CMR), for example, when examining COVID-19 convalescents using magnetic resonance imaging (MRI), has an influence the exposure profiles of medical personnel to static magnetic fields (STmf). (2) Methods: Static exposure to STmf (SEmf) was recorded during activities that modelled performing CMR by radiographers. The motion-induced time variability of that exposure (TVEmf) was calculated from SEmf samples. The results were compared with: (i) labour law requirements; (ii) the distribution of vertigo perception probability near MRI magnets; and (iii) the exposure profile when actually performing a head MRI. (3) Results: The exposure profiles of personnel managing 42 CMR scans (modelled using medium (1.5T), high (3T) and ultrahigh (7T) field scanners) were significantly different than when managing a head MRI. The majority of SEmf and TVEmf samples (up to the 95th percentile) were at low vertigo perception probability (SEmf < 500 mT, TVEmf < 600 mT/s), but a small fraction were at medium/high levels; (4) Conclusion: Even under the “normal working conditions” defined for SEmf (STmf < 2T) by labour legislation (Directive 2013/35/EC), increased CMR usage increases vertigo-related hazards experienced by MRI personnel (a re-evaluation of electromagnetic safety hazards is suggested in the case of these or similar changes in work organisation).

## 1. Introduction

Magnetic resonance imaging (MRI) is currently the gold standard for evaluating cardiac morphology and function [1]. Recent cardiac magnetic resonance (CMR) mapping techniques, including T1, T2 and extracellular volume, are unique tools for quantitative assessments of myocardial diffuse fibrosis and edema [2]. The ongoing COVID-19 worldwide pandemic caused by the SARS-CoV-2 coronavirus (with the number of cases still rising rapidly worldwide—from over 1 million cases in April 2020 to over two hundred million by August 2021) has affected nearly every aspect of life, at least during 2020 and 2021, but it has not stopped MRI diagnostics, even of infected patients [3,4].

COVID-19 mainly affects the respiratory system; however, due to the increasing number of infections, an growing number of accessory morbidities, such as cardiac or nervous system damage, are also being diagnosed. Consequently, the scope of examinations performed using MRI scanners in many diagnostic units has recently changed significantly due to the rapid growth of specific clinical conditions that were not as commonly diagnosed before the pandemic. According to published studies [5,6], up to 15% of patients with COVID-19 had elevated high-sensitive cardiac troponin I (hs-cTnI) during hospitalisation, an indication of myocardial injury, and cardiac involvement in severe-type patients was up to 31%. Cardiac involvement in various types of myocarditis, including myocardial fibrosis, edema, and pericarditis [7], is associated with poor prognosis; it is important to identify it at an early stage in order to administer the appropriate treatment. Because of this, expanded usage of CMR and an increased need for cardiac diagnostics of COVID-19 convalescents has been observed.

In regular practice before the COVID-19 pandemic, CMR was performed only in highly specialised diagnostic units, mostly to scan the head, spine, abdomen and pelvis (e.g., in our MRI unit during March–June, 2019, these represented 8, 23, 37 and 23% of all scans, respectively). The majority of regular outpatient care MRI units did not use CMR at all, or only rarely, instead performing almost exclusively head MRI scans or a mixture of head and similar spine scans. Because of this, regular MRI practice may be considered to be nearly equivalent to performing head MRI scans almost exclusively. COVID has led to the increased use of CMR in highly specialised diagnostic centres (for example, CMR use increased by up to 45% during March–June, 2020 in our unit) and its broader use in other centres.

Forecasts for the evolution of the pandemic and its health consequences justify the expectation that, at least in the near future, the healthcare system and work practice will also face the need to manage the delayed health consequences of infections. The discussed dynamic changes in CMR usage and its increased use related to the COVID-19 pandemic are expected to affect many MRI units worldwide. The developed experience in CMR may increase its usage, also when pandemic-related health problems decrease in future.

### 1.1. The Profile of EMF Exposure Related to MRI Usage

MRI images are obtained by exposing patients (inside a magnet bore) to electromagnetic fields (EMF) during diagnostic procedures (the acquisition of scans), i.e., static magnetic field (STmf), radiofrequency and gradient fields. Patients entering an MRI scan room are assisted by radiographers or nurses in taking the proper position on the MRI table, and are prepared for the examination or helped when they are exiting the table and leaving the room after the examination [8,9]. A CMR procedure also includes the administration of a contrast medium or cardiac pharmaceuticals to the patient. Usually, when the acquisition of scans has stopped, the administration of such pharmaceuticals is performed with medical assistance by a nurse or a cardiologist. Alternatively, pharmaceuticals are administered by infusion pump during the acquisition procedure. Typically, it is not common for radiographers, nurses and clinicians (cardiologists, anaesthesiologists or radiologists) to stay with patients while scans are being taken, so they may generally be exposed to STmf only, i.e., they are not affected by the radiofrequencies and gradient fields used to scan patients [8,9,10,11,12,13,14,15].

The activities of MRI healthcare personnel (usually outside the magnet bore) result in: (i) static (actual level) exposure to spatially inhomogeneous STmf (SEmf) near the permanently active magnets of MRI scanners, and (ii) motion-induced time-variability in STmf exposure level (TVEmf). Both these components are related, in a highly complex way, to the level of the main field of the scanner and the spatial distribution of STmf in the vicinity of the magnet, the needs of a particular patient, work practices and the equipment in the MRI unit. They should be evaluated separately in terms of occupational safety requirements [16,17,18,19]. Healthcare personnel approaching only a magnet are affected by an STmf which is at least 30% weaker than the main field affecting the patient inside the bore of the magnet [10].

### 1.2. EMF Safety Issues near MRI Scanners

The STmf near the magnet housing is strong enough to cause various health and safety hazards (even dangers to life) due to its influence on humans and other objects. Among the best-known hazards are flying objects (projectile hazards from ferromagnetic objects) and malfunctions in electronic devices with insufficient immunity to STmf and time-varying EMF influence; both aspects require prudence in electromagnetically shielded scan rooms [16,19,20]. Another well-known health and safety hazard is the stimulation of sense organs in humans moving across strong spatially inhomogeneous STmf, sometimes experienced by MRI personnel, for example in the form of vertigo or the loss of balance perception (which may trigger related safety hazards or accidents caused by disturbances in the performance of their work) [16,18,19,21,22,23,24,25,26,27,28,29,30]. The mentioned stimulation of sense organs may be experienced by MRI personnel only when they are approaching or entering the magnet’s bore, where the STmf is significantly stronger than the threshold for projectile hazards. That hazard is significantly more localised near the magnet’s bore compared to the flying objects hazard, which affects nearly the entire scan room.

### 1.3. The Aim

The purpose of our study was to evaluate the impact of EMF associated with the frequent application of CMR, i.e., of medium (1.5T), high (3T) and ultrahigh (7T) fields, on the safety of healthcare personnel (in particular, radiographers).

This study evaluates the hypothesis that the significant increase in CMR use observed recently, largely due to the diagnostic needs of patients who have recovered from COVID-19, also has a significant influence on the profiles of STmf exposure of MRI personnel, which may have an impact on vertigo-related safety hazards experienced inside scan rooms.

## 2. Materials and Methods

### 2.1. Characterisation of the Workplace and Worker Tasks

Prior to the experimental part of this study, the analysis of the MRI work environment covered an inventory of: (i) MRI accessories used for various examinations; (ii) the spatial management of particular accessories in the scan room; and (iii) the spatial distribution of STmf near MRI magnets.

A structured questionnaire regarding the list of accessories used for various types of MRI scans (independently of the scanner type) was completed voluntarily by experienced MRI personnel. Six radiographers with over 15 years of experience in MRI in various highly specialised diagnostic units equipped with scanners from various vendors were asked to give their opinions, as were the service engineers collaborating with our MRI unit. They were also queried about the routine tasks and movements inside the scan room performed by radiographers, nurses and cardiologists. A set of typical accessories used for various MRI scans was identified. A set of typical accessories used for CMR was also modelled using a set of volume non-metallic models with dimensions which were compatible with particular accessories, together with a relevant model of a patient which was fully autonomous (regarding physical activity). In addition, the scenario of the relevant location of a particular model at the table or relative to the patient’s body was identified, along with trajectories of movements when radiographers are preparing the scanner and necessary accessories, assisting the patient before and after the examination, and replacing the accessories before and after the CMR.

Together, we created a model of a single “radiographer exposure event” (REE) to inhomogeneous STmf inside an MRI scan room, i.e., the activities of a radiographer who is performing a set of complex tasks associated with a typical CMR scan of a single autonomous patient being affected by STmf near an MRI magnet. The parameters of exposure to STmf during REEs are analysed further in this paper. REEs should not be taken as representative for exposure by practitioners of other professions, such as cardiologists, nurses or cleaners.

Although CMR involves pharmaceutical injections (mostly contrast) more frequently than regular MRI, the exposure of nurses to STmf was not considered in detail in our study based on the assumption that the organisation of the relevant tasks are comparable to those discussed in detail in a previous paper [10]. We were unable to collect relevant information to consider the details of cardiologists’ exposure while performing CMR. In short, cardiologists often supervise CMR sessions while staying in the scan room near a patient, or from a computer console located elsewhere (in the control room). It also needs to be pointed out that neither assistance given to disabled patients, whereby more complex tasks are performed by radiographers, nor the activities of cleaners, were considered in detail in this study because of limited access to MRI units related to pandemic restrictions.

### 2.2. Measurement Strategies and Data Collection

This study was based on measurements using a vector STmf-sensitive Hall probe. The spatial distribution of exposure near magnets was characterised by the results of spot measurements of STmf absolute values (i.e., magnetic flux density, B, expressed in militeslas, mT; also known as the B-field).

Additionally, B-field samples actually affecting investigators were recorded by a real-time data logger (described as an “exposimeter” hereafter) throughout their movements mimicking a radiographer’s activities near the magnet, to characterise SEmf in a particular REE. The investigators performing the reported study followed a defined path inside the scan room with a Hall sensor, connected to a pocket data logger, attached to their chest or head. The modelled radiographer exposure was recorded by an exposimeter carried by an investigator while performing a defined series of tasks related to moving various items from among the prepared set of models representing the MRI accessories used to perform cardiac MRI, as well as the model of a patient. Volume models of MRI accessories and the model of the patient’s body were relocated in a realistic way between the control room, the storage area and the MRI table while measurements of exposure were taken during each REE.

The motion-induced time variability of exposure level (TVEmf) was derived from the collected SEmf samples (dB) and the time associated with them (dt). Both measurements were performed using a THM-1176 data logger, Metrolab Technology SA, Geneva, Switzerland, with a resolution of ±0.5mT, a measurement range of 0.1–20,000 mT and a 6–25 Hz sampling rate. Calibration at the 2% uncertainty level (in the CIOP-PIB laboratory, certificate No AP061) and a zero-field adjustment of the sensor were carried out prior to data collection.

This study was carried out without recording personal data of workers, adhering to the labour law regulations for work in environment with EMF sources whereby no human or animal studies were involved. Written agreement with the supervisors of the MRI units was obtained before implementing the study procedure. Additionally, experiments related to the development of diagnostic potential of the ultrahigh 7T MRI scanner were supervised by the local bioethical commission, which gave approval for these activities.

### 2.3. Statistical Analysis

The results of measurements were characterised by the statistical parameters of sets of STmf samples recorded during individual REEs (using STATISTICA 10.0 software, StatSoft, Palo Alto, CA, USA). The significance of differences between REEs were tested using the ANOVA Kruskal–Wallis nonparametric test (significance level: *p* = 0.05) for independent groups that may not have a normal distribution, because the analysis showed that the distribution of samples in REEs was not normal (a Shapiro-Wilk test; significance level: *p* = 0.05).

### 2.4. STmf Metrics and Evaluation Rules

In line with common practice, the orthogonal components of the B-field (Bx, By, Bz) were recorded at the same time-points (t), and used to calculate the absolute values of B vector (i.e., SEmf) and dB/dt (i.e., TVEmf) [11,12,13,14,15,18,25,27,28,29]:

(1) $$B=\mathrm{SEmf}=\;\sqrt{{\left(\mathrm{Bx}\right)}^{2}+{\left(\mathrm{By}\right)}^{2}+{\left(\mathrm{Bz}\right)}^{2}}$$

(2) $$\frac{\mathrm{dB}}{\mathrm{dt}}=\mathrm{TVEmf}=\;\sqrt{{\left(\mathrm{dBx}/\mathrm{dt}\right)}^{2}+{\left(\mathrm{dBy}/\mathrm{dt}\right)}^{2}+{\left(\mathrm{dBz}/\mathrm{dt}\right)}^{2}}\;$$

The obtained parameters of SEmf and TVEmf were compared with:

*(1)*  *The distribution of the probability of vertigo perception under STmf exposure.*

We analysed the results of studies regarding subjective reports on the perception of vertigo during the regular work of MRI healthcare personnel before the COVID-19 pandemic, as well as those on the distribution of the probability of this vertigo perception versus various independent parameters of exposure to STmf, as provided by Schaap et al. [25]. Based on these results, we developed a simplified system of categorisation regarding the probability of hazards associated with vertigo perception near MRI magnets using a three-level scale (low, medium and high level of vertigo perception probability) according to various exposure metrics (SEmf and TVEmf), assessable by exposimetric measurements (see Table 1). Levels of exposure metrics associated with various categories of vertigo perception probability (VP) were evaluated based on VPS, i.e., the vertigo perception probability published by Schaap et al. [25], using the following criteria: low VP (LVP) was set at VPS < 10%; medium VP (MVP) at 10–90%; and high VP (HVP) at VPS > 90%.

*(2)*  *The limits of STmf exposure set by relevant international guidelines and legislation.*

With respect to exposure to STmf and its variability caused by the movements of humans near the magnet, the European Directive 2013/35/EC [19] to prevent sensory and health exposure effects (Table 1) was used as the basis for our exposure limit values (ELVs).

The sensory effects considered regarding ELVs were defined as the effects of exposure to STmf, such as perception of vertigo and other physiological effects related to disturbances in the human balance organ, as well as effects from the induced electric field on the central nervous system in the head, such as retinal phosphines and minor transient changes in brain function [19]. The ELVs regarding sensory effects are provided for an evaluation of EMF exposure under “normal working conditions” (NWC), regarding tasks performed in conformity with the relevant labour laws.

Health effects with respect to ELVs were defined as the effects of exposure to STmf, such as the electric stimulation of all peripheral and central nervous system tissues in the body, including the head [19]. ELVs regarding health effects may only be applied to evaluations of STmf exposure among workers on a temporary basis during the shift, and only when exposure to STmf is justified by the practice and process, provided that preventive measures, such as controlling movements and providing information to workers regarding exposure and its possible effects and related hazards, have been adopted, constituting “controlled working conditions” (CWC) [19].

ELVs are expressed in the B-field in the workplace (in the 0–1 Hz frequency range) and in the electric field induced by the time-variability of exposure to STmf in the body of an exposed worker (in the 1 Hz–10 MHz frequency range) [18,19]. Evaluations of exposure near sources of STmf should consider the 0–25 Hz frequency range [17,18]. Compliance of exposure with the ELVs set for the B-field parameters may be directly evaluated by measuring the B-field (spot or exposimetric measurements of SEmf). However, direct evaluations of compliance with the ELVs set for the electric field induced in the body are not achievable, as they involve numerical simulations of the induced electric field, or an evaluation of substitute parameters of exposure with equivalent limits (i.e., reference levels (RL)) regarding the time derivative dB/dt–converted from limits set for exposure to sinusoidal B-fields in the workplace, i.e., an evaluation of TVEmf [18].

*(3)*  *Exposure profile in regular MRI practice.*

The profile of STmf exposure during regular MRI practice was considered based on the results of previous studies regarding exposure among radiographers while performing the most common MRI scans (head and spine scans, which are similar in both their procedure and the MRI accessories used), as shown in a previous paper [26]. The regular MRI activities of radiographers were characterised by REEs collected previously using an exposimetric measurement method comparable to the one used in the reported CMR studies.

## 3. Results

### 3.1. MRI Accessories

**Figure 1:**
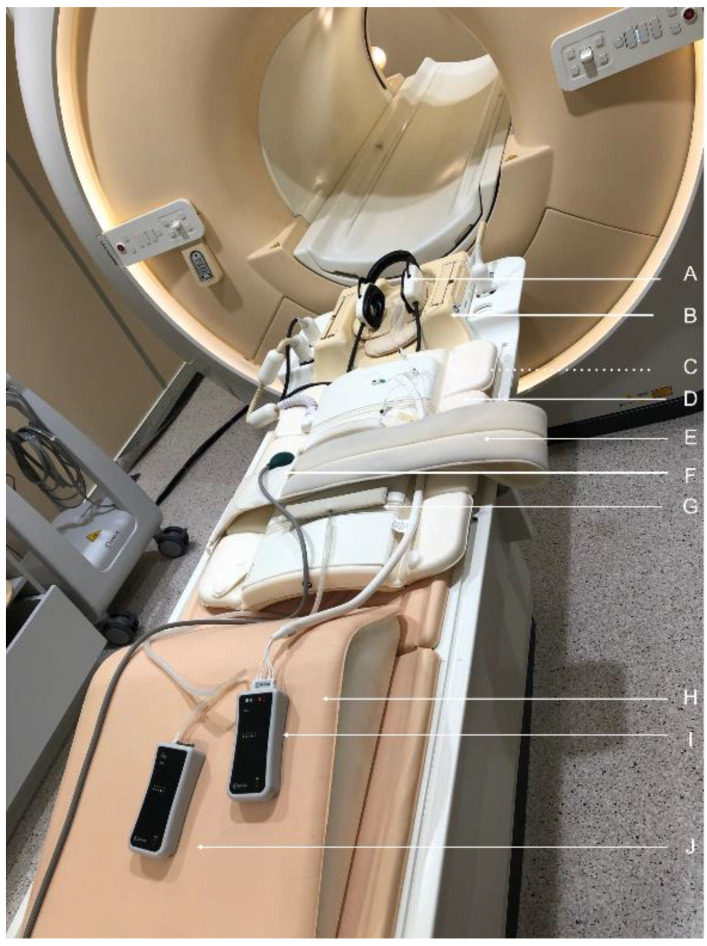
A set of MRI accessories usually used in CMR procedures, located on the MRI table following the usual order when a patient is prepared for an examination: (i) ear protection/headphones, EP, (**A**) located at the lower part of two-piece head coil, TP-C, and head stabiliser, HS, (**B**); (ii) cardiac monitor, CM, electrodes (**C**); (iii) body surface coil/flexible, BS-C, (**D**) and a stabilising strap (**E**); (iv) communication ball, CB, (**F**); (v) respiratory monitor, RM, sensor (**G**); (vi) lower limb stabiliser, LLS, (**H**); (vii) CM module (**I**); (viii) RM module (**J**).

The typical use of accessories in adult MRI scans was characterised based on the outcomes of the aforementioned questionnaire and consultations (Table 2). The set of 9–10 accessories usually used in CMR procedures is shown in Figure 1. In contrast, MRI procedures on the head (recognised here as constituting the majority of regular MRI practice) typically involve only four accessories (Table 2).

### 3.2. STmf near MRI Magnets

**Figure 2:**
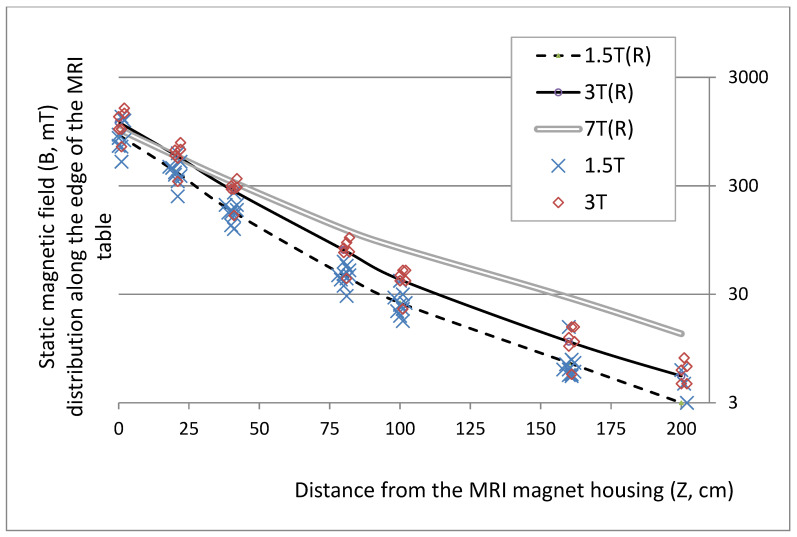
Plot of static magnetic field distribution along the edge of the MRI table, derived from the results of spot measurements of magnetic flux density (B, in mT) near 16 whole-body, closed-bore, horizontal-field, MRI magnets (symbols represent data regarding individual magnets, and lines represent results averaged over groups of magnets: 10 MF-MRI (1.5T) magnets, five HF-MRI (3T) magnets and one UHF-MRI (7T) magnet). Note to editors: please find copies of all figures in enclosed files, please keep the caption together with a figure on the same page.

The distribution of STmf was studied near 16 typical MRI scanners, representing the various types used in Europe, i.e., whole body, closed bore, horizontal field magnets, as follows: 10 medium 1.5T field magnets (MF-MRI) of GE Signa (Creator and Infinity), Philips (Achieva and Ingenia), Siemens Magnetom (Essenza, Aera, Avanto, Avanto Fit and Symphony) and Toshiba (Vantage Atlas); five high 3T field magnets (HF-MRI) of GE Signa Architect, Philips (Achieva and Ingenia) and Siemens Magnetom (Trio and Skyra); and one ultrahigh 7T field magnet (UHF-MRI) GE. The STmf distribution along the edge of the MRI table is shown in Figure 2. The STmf side to the magnet cover may be considered negligible with respect to the aforementioned vertigo-related hazards from STmf exposure.

### 3.3. Radiographer Exposure to STmf

**Figure 3:**
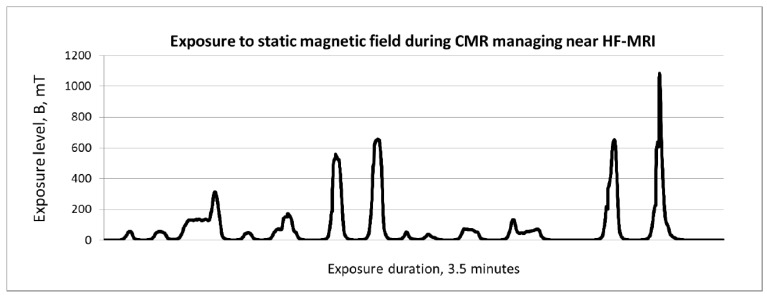
Example of the results of SEmf measurements during a modelled radiographer exposure event (REE) related to CMR in an HF-MRI scanner, managed by an investigator equipped with an exposimeter with a Hall probe located on the chest (C). Note to editors: please find copy of all figures in enclosed files, please keep the caption together with the figure on the same page.

SEmf measurements during each modelled radiographer exposure event (REE) were collected (Figure 3). As shown in Table 2, the following tasks were included in each modelled REE: (i) moving the lower part of a two-piece head coil from a storage space to the table (close to the magnet), (ii) assisting a patient entering the scan room and taking the proper position on the table, (iii) moving a cardiac monitor, a respiratory monitor, a communication ball and ear protection from the storage space and locating it on the patient’s body, (iv) covering the patient with the upper part of the head coil and body surface coil (and fixing it with stabilising straps), and (v) inserting the table into the magnet bore using a manual console on the magnet cover. The second part of the REE covered exposure when removing all the accessories from the table and assisting the patient in leaving the table and the scan room. Taking into account specific hygiene procedures implemented because of the COVID-19 pandemic, disinfections of the table and the magnet entrance were carried out at the beginning of each REE. Overall, 42 REEs related to the modelled CMR performance were recorded in close proximity to the human whole-body actively shielded scanners: MF-MRI (1.5T), HF-MRI (3T) and UHF-MRI (7T). The analysed REEs were collected; this involved three investigators performing the aforementioned activities modelling CMR. The investigated CMR were modelled considering only activities needed when (physically autonomous) patients receive standard scans.

To compare CMR and the most typical MRI scans, the same parameters for STmf exposure during REEs were derived from exposimetric measurements performed during the real work of radiographers with 13 patients receiving the most common head and spine scans in various MF-MRI scanners. These results have been analysed and characterised in a previous paper [26]. The characteristics of exposure while performing regular MRI procedures included assisting able-bodied patients, as well as one case of assisting a disabled patient.

The SEmf and TVEmf samples were characterised by the statistical parameters shown in Table 3: (i) parameters of samples in a single REE; (ii) parameters of REEs split into subsets of REEs collected near a single magnet type; and (iii) parameters of all samples recorded near a single magnet type (considered as a single combined set of samples).

We decided to combine REEs, including measurements using an STmf probe located at the head and chest of the investigators performing modelled CMR, into single subset of REEs recorded near a particular magnet type (i.e., medium, high, or ultrahigh field), because only approximately 50% of the analysed pairs of REEs recorded near each scanner type were statistically significantly different compared to each other (including both locations of the probe) (Kruskal-Wallis tests). Up to the 99th percentile of the analysed samples in REEs, these differences in the results were most significant in data collected near UHF MRI (up to 25% only); only in the maximum values of both parameters was head exposure stronger. This is because of the vertical distribution of STmf near the magnet, i.e.,where head exposure is significantly lower than torso exposure, balancing the faster movements of the head.

**Figure 4:**
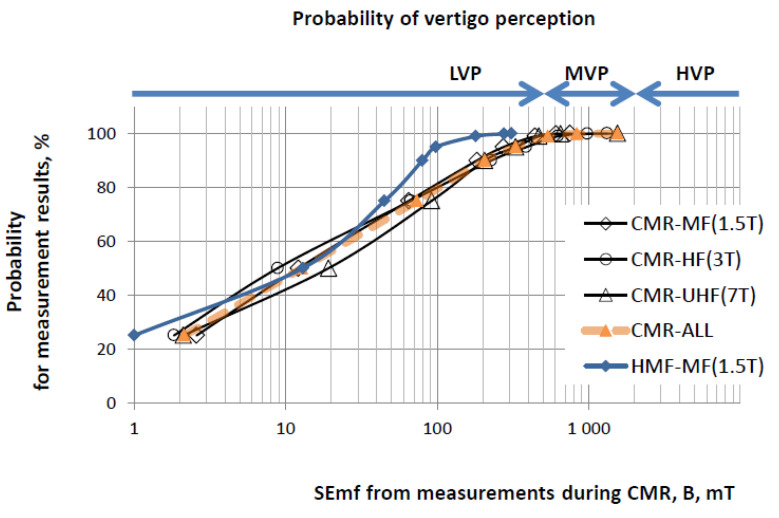
Distribution of the results of measurements collected by an exposimeter during the performance of 42 modelled CMR and 13 real head MRI radiographer exposure events (REEs). Static exposure to the magnetic field near MRI magnets, SEmf in mT, and a relevant evaluation of the probability of vertigo perception due to such exposure (LVP, MVP and HVP as shown in Table 1 regarding a peak value over the shift, PB@s).

**Figure 5:**
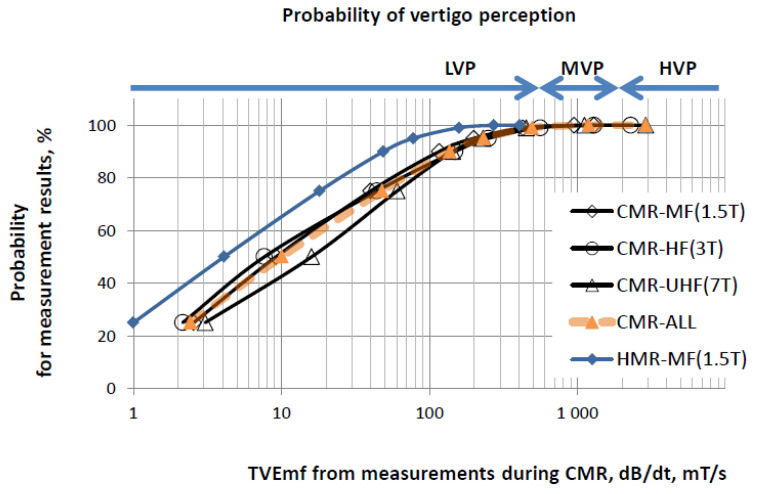
The distribution of the results of measurements collected by an exposimeter during the performance of 42 modelled CMR and 13 real head MRI radiographer exposure evenats (REEs). Time-variability of exposure caused by movements near MRI magnets, TVEmt in mT/ s, and relevant evaluation of the probability of vertigo perception at such exposure (LVP, MVP and HVP as shown in Table 1 regarding a peak value over the shift, PdB/dt@s).

The recorded SEmf showed that the modelled activities for managing CMR (in 1.5T, 3T and 7T scanners) and the real head MRI scans (in 1.5T) were performed in locations where the levels of STmf were compliant with ELVs regarding sensory effects (B < 2000mT), as mentioned in Table 1 (Figure 4). Following the provisions of Directive 2013/35/EU, such exposure is classified with respect to the STmf exposure of workers under NWC. However, according to our measurements, the TVEmf wa at the NWC level even during fast movement (dB/dt < 600 mT/s, set at 3 Hz as the RL limit derived from relevant ELVs regarding sensory effects; see Table 1), but only while managing the head MRI scans in a 1.5T scanner. When performing CMR scans, a small fraction of recorded samples (<1%) obtained TVEmf values reaching the RL limit designated as “controlled working conditions” (CWC) regarding sensory effects for fast movement in performing tasks in STmf in the workplace (dB/dt > 600 mT/s). Near 7T scanners may even reach the RL limit of TVEmf set for CWC regarding sensory effects for slow movement and health effects for movement of any speed (dB/dt > 2700 mT/s), (Table 1, Figure 5).

In each CMR-related REE, the majority of SEmf and TVEmf samples were at levels associated with a low probability of vertigo perception (at least up to the 95th percentile of samples). However, in the remaining small fraction, the recorded levels of exposure (3–5 times higher SEmf and 6–12 times higher TVEmf) were associated with a medium or even high probability of vertigo perception (Table 3). In addition, the time-averaged parameters of exposure over REEs (Aver) also indicated a medium or even high probability of vertigo.

If REEs during MRI scans of disabled patients are excluded, the duration of REEs for a head MRI is much shorter than that shown in Table 3 (170/72-410), i.e., on average, 100 s, within a range of 72 to 210 s. Exposure recorded for a head MRI (1.5T) indicated a low probability of vertigo. The duration of a particular REE is comparable between the analysed subset. It must be stated that recorded exposure related to a CMR scan was longer than in a head MRI, even when taken as an average (i.e., when scans of active and disabled patients were considered together). Compared to head MRI scans of active patients only, the duration of exposure related to a CMR scan is nearly twice as long. However, it must be pointed out that even the longest duration of REEs is in the order of 4 minutes, which is about 10% of the duration of a single CMR scan. As such, there is a low probability that the increased use of CMR may significantly influence the number of patients diagnosed daily in an MRI unit.

The profile of SEmf recorded over 12 modelled CMR with a medium field scanner (1.5T) was significantly different (Kruskal-Wallis tests) from exposure in regular MRI practice (1.5T).

Within our study, the distribution of vertigo perception probability associated with STmf exposure parameterised by the peak (actual) value of B or dB/dt during a shift (as studied by Schaap et al. [25]) was compared with the values of SEmf or TVEmf samples recorded during the considered radiographer exposure events (REE), i.e., exposure while managing the MRI scan of a single patient. The profile of the distribution of vertigo perception probability against the values of B or dB/dt, which were time-averaged over exposure inside the scan room (in line with the methods of Schaap et al. [25]) were compared with the relevant values averaged over the total duration of the analysed set of considered REEs. This is compatible with a case when, during a shift, only the analysed CMR scans are managed by radiographers, i.e., mimicking a possible exposure situation reflecting the worst case of the influence of increased CMR performance.

We did not consider exposure throughout the entire shift, as this study was focused on the parameters of exposure from the perspective of an individual REE, which are not dependent on the number of patients per day. For the case of a particular mode of organisation of an MRI service in a particular MRI unit, the shift-averaged parameters of exposure may be analysed using our data when the real number of scans and the duration of shifts are known. As far as we are aware, different MRI units organise their work differently, and the results of such analyses may be very individualised, whereas the characteristics of exposure per individual REE, as discussed in our study, are more general.

## 4. Discussion

### 4.1. Significance and Limitations

The distribution of STmf along the edge of table indicates that exposure among radiographers at a short distance from the magnet (SD of 0–50 cm mentioned in Table 2) may be more than four-times that at a medium distance (MD of 50–100 cm), while at a long distance (LD over 100 cm away from the magnet), exposure may be considered negligible.

CMR involves the highest number of accessories, i.e., up to 10, of which at least nine need to be manually managed at the table and by the patient, whereas previous and subsequent scans are different from CMR (with four needing to be managed at a short distance from the magnet, where STmf is at the highest level and has the greatest spatial heterogeneity). In contrast, the most widely-used head MRI scans may involve only four accessories (of which three are operated at a short distance from the magnet). In our study, STmf exposure was recorded during the modelled unified scenario (i.e., changing the positions of accessories and assisting the patient model to enter or leave the scan room or get off the MRI table after the CMR) and applied equally to all scanners, despite variations in design and the needs of real patients. This strategy of measurement and data collection justified the comparative analysis of REE parameters using MRI scanners of various magnet types (medium, high and ultrahigh field). We believe that our exposure scenario recreated worst-case REEs, as realistic situations may reduce the average duration of exposure and the probability of short but strong exposure (such as that resulting from the use of accessories integrated into the table or magnet structure, or the performance of consecutive CMR scans without the need to replace accessories).

However, it must be said that in a small fraction of scans, stronger exposure than that recorded in our study may occur, e.g., when disabled patients need more attention, thereby creating longer exposure times (as shown in Table 3), or when personnel enter the bore of the magnet for any technical or organisational reason, where the main field is many times stronger than those recorded during the modelled procedure (2–4 times higher compared to the maximum recorded in our study SEmf, but 5–20 times higher when compared to the 95th centile of measurement results; 5.5 times higher near 1.5T magnets, 7.7 times higher near 3T magnets and 20 times higher near 7T magnet), especially inside HF-MRI and UHF-MRI magnets (3T and 7T, respectively).

Because of this, it must be stated that the requirements provided by labour law regarding CWC with respect to STmf in the workplace need to be applied to any activities inside an HF-MRI and UHF-MRI scan room, because the levels of their main fields exceed 2T (i.e., exceeds the level of ELV regarding Sensory Effects) inside the magnet bore, which is accessible by healthcare personnel, even workers rarely enter the main field in regular practice).

The differences between exposure parameters near various scanners were smaller than expected (i.e., while approximately 50% of statistically significantly different cases were found between REEs recorded on particular scanners, less than 80% of REEs were significantly different between various scanners; Kruskal–Wallis test, *p* < 0.05). This supported our hypothesis that, by using equal exposure scenarios, exposure to STmf at each scanner is well represented. This relatively low variability may be explained by the small differences in the distribution of STmf away from the magnet along the table (i.e., much smaller than in the levels of the main field of particular magnets, as shown in Figure 3). This finding was not surprising when we consider that the strongest magnet (used in the ECOTECH-COMPLEX Research Centre, Lublin) is a next generation superconducting Ultra High Field (7T) large bore, a whole-body magnet of large homogeneous field volume with an active shield zero boil-off (produced by Tesla Engineering Ltd., UK).

We expect that variations in REEs in real MRI practice may be larger, but due to pandemic restrictions hindering access to healthcare units, it is impossible to confirm this at present.

A larger variability of scan types is possible when stronger magnets are used. Taking this into account, our data indicate that the differences in parameters characterising the exposure of radiographers (during work with real patients over the course of their shifts, with various 1.5T or 3T scanners) found in other studies may be explained by differences in workplace practices (including variability of the scan types performed during a shift), as opposed to the proposals of authors who only considered different levels of the main field in the scanner bore [25,28,31]. All this supports the need to perform broader investigations into the discussed problems when the pandemic is over, as well as with respect to the various factors influencing the applications of MRI.

### 4.2. Application Potential

The needs to evaluate the probability of vertigo perception in the MRI workplace, and to implement adequate safety measures in order to decrease hazards related to improperly performing the necessary procedures (dangerous for workers and patients) are underlined in safety guidelines and are formally required by binding European labour laws [18,19]. This is because at least several per cent of MRI workers were found to be susceptible to vertigo and other symptoms caused by STmf. Symptoms may be unexpected and severe enough to cause safety hazards to both workers and patients, and may also negatively affect the ability to work. The sensitivity of humans to SEmf is highly individualised and, contrary to the aforementioned influence on material objects, less systematically characterised in safety guidelines and labour law requirements [16,17,18,19,21,22,23,24,25,26,27,28,29,30,31].

European Directive 2013/35/EU formally entered into force in 2016, so we assume that in well-organised MRI units, these hazards are already being managed. However, as regular practices change, the probability of experiencing vertigo may also change and needs to be re-evaluated and updated, as required by relevant European legislation, i.e., Articles 4.7 and 5.9 of Directive 2013/35/EU [18]. In this context, our study provides a method of systematic analysis of the impact from any changes in work practices in MRI diagnostic units. It was used, for example, to analyse the impact of the recently observed increased use of CMR for COVID-19 convalescents. However, our approach and the developed characteristics of MRI accessories usage (Table 2) may easily be adopted to analyse any other impact of exposure among healthcare personnel to STmf and related occupational health and safety hazards, arising from various changes in work practices caused by other factors, such as the use of new accessories or the provision of services for new health problems.

### 4.3. Further Research Directions

Given our observation that the parameters of radiographer exposure to STmf are associated with a higher probability of vertigo perception during exposure related to CMR than in regular MRI practice, it should be pointed out that higher sensitivity to vertigo was found during the first period of employment in an MRI unit [31]. If this is the general case, the increased probability of vertigo perception expected from changes in the work practice in an MRI unit may also only be temporary. This hypothesis needs verification through experiments.

We believe that our investigations may help MRI units to update their occupational health and safety standards to accommodate for changes in the work organisation, such as the aforementioned changes resulting from the COVID-19 pandemic or other circumstances. Our investigation method involving the evaluation of STmf exposure while working with models of MRI accessories and models of patients may easily be used to promote the application of good work practices in MRI units. It may also be used to test the influence of modifications to work practices on exposure to STmf among personnel, and the risk of accidents or malfunctions related to the perception of vertigo and loss of balance experienced by workers.

## 5. Conclusions

Because of current pandemic restrictions regarding access to MRI units, our study was limited in scale. However, our intention was to focus on the most important aspects of the analysed problem by modelling relevant work practices without contact with patients.

Systematic analyses of the parameters of modelled MRI static and dynamic exposure to STmf by radiographers were performed with respect to the variability of MRI accessories and the level of the main field inside the bore of the actively shielded magnet (1.5T, 3T and 7T) of a given MRI.

The results confirmed our hypothesis that increased CMR usage (or similar changes in work organisation) needs to be followed by a re-evaluation of electromagnetic safety hazards (as required by the labour law), because this increases the probability of perception by MRI personnel of vertigo, as well as exposure to related health and safety hazards, compared to regular MRI practices (including the common performance of head MRI scans), even when work activities are carried out in an environment that was previously classified as constituting “normal working conditions”, as defined by Directive 2013/35/EC with respect to STmf affecting workers (namely, when the work is performed in an STmf not exceeding a level of 2T).

## Figures and Tables

**Table 1:** Criteria for evaluating exposure among healthcare personnel to static magnetic fields near MRI scanners.

Exposure Type	Units	Exposure Metrics	Level of Exposure Associated with Various Categories of Vertigo Perception Probability (VP) [25]			Exposure Limits Provided by International Legislation and Guidelines	
			LVP <10%	MVP 10–90%	HVP >90%		
SEmf, B	mT					Exposure Limit Values (ELVs) provided by European labour law, Directive 2013/35/EU [19]	
						Sensory Effects ELVs for NWC	Health Effects ELVs for CWC
		PB@s	500	~1000	2000	2000	8000
		TAB@r	75	~150	300	---	---
		TAB@s	4	~7	12	---	---
TVEmf dB/dt	mT/s					Limits (Reference Levels) of TVEmf derived from relevant ELVs regarding [18]:	
						Sensory Effects	Health Effects
		PdB/dt@s	600	~1000	2000	@0.5 Hz: 2700 @1.0 Hz: 1800 @3.0 Hz: 600	@0–25 Hz: 2700
		TAdB/dt@r	6	~20	50	---	---
		TAdB/dt@s	0.7	~1.1	1.4	---	---
Notes: SEmf—static exposure to a magnetic field (actual value of exposure recorded by exposimeter), B-field PB@s—peak value of B during a shift, i.e., the maximum value of SEmf samples in all REEs during a given shift TAB@r—time-averaged value of B for exposure inside the scan room only, i.e., the averaged value of SEmf samples in all REEs during a shift TAB@s—time-averaged value of B during a shift, i.e., time-averaged parameter of exposure which is sensitive to the number of patients and the duration of a given shift TVEmf—time-variability of exposure to the magnetic field (motion-induced value calculated from SEmf samples), dB/dt derived from SEmf samples PdB/dt@s–peak value of dB/dt during a shift TAdB/dt@r—time-averaged value of dB/dt over exposure inside the scan room only TAdB/dt@s—time-averaged value of dB/dt over the shift Probability of vertigo perception: LVP–low (<10%); MVP—medium (10–90%); HVP–high (>90%). Working Conditions: NWC–Normal; CWC–Controlled The limits regarding TVEmf, which were derived from the relevant sensory effects of ELVs for the electric field induced inside humans (reference levels), are frequency dependent: 2700 mT/s @ 0–0.66Hz, (1800/f) mT/s @ 0.66–8Hz and 220 mT/s @ 8–25Hz, where f is the frequency expressed in Hz. In contrast, the limits regarding TVEmf, which were derived from the relevant health effects ELVs set for electric fields induced inside humans, were over the 0–25 Hz frequency band (Table 1) [18].							

**Table 2:** Typical MRI accessories used for the most typical adult MRI diagnostic procedures.

Examination Type	MRI Accessories											
	1.TP-C	2.OP-C	3.IP-C	4.BS-C	5.CM	6.EP	7.CB	8.RM	9.HS	10.LLS	11.CAM	**12.CAA**
Head MRI	X	O	nu	nu	O	X	X	nu	X	O	O	O
Cervical spine	X	O	X	nu	O	X	X	nu	X	O	O	O
Limbs (joints)	nu	X	O	O	O	X	X	nu	nu	nu	O	O
Thoracic	nu	O	X	X	O	X	X	nu	O	X	O	O
Lumbar spine	nu	nu	X	nu	O	X	X	nu	nu	O	O	O
Abdomen and pelvis	nu	nu	X	X	O	X	X	O	nu	O	X	O
Cardiac MRI	O	nu	X	X	X	X	X	O	X	X	X	O
Notes: MRI accessories/the distance from the MRI magnet where it is manually managed at the MRI table or the patient’s body: short (SD), i.e., <50 cm; medium (MD), i.e., 50–100 cm; long (LD), i.e., >100 cm: 1. TP-C/(SD)—two-piece head coil (birth cage type) 2. OP-C/(SD)—one-piece coil 3. IP-C/(MD)—posterior coil (no manual operation needed when integrated with diagnostic table) 4. BS-C/(MD)—body surface coil (flexible) 5. CM/(MD)—cardiac monitor–electrodes and module 6. EP/(SD)—ear protection (earplugs/head phones) 7. CB/(MD)—communication ball 8. RM/(MD)—respiratory monitor–sensor and module 9. HS/(SD)—head stabiliser 10. LLS/(LD)—lower limb stabiliser 11. CAM/(SD)—contrast administering staff; manual 12. CAA/(SD)—contrast administering staff; automatic The use of MRI accessories: X—typically used in a particular MRI examination type; O—optional use, depending on the details of a particular MRI examination case or MRI scanner type; nu—not used in a particular MRI examination type												

**Table 3:** Exposure to STmf related to CMR and head MR activities of radiographers near various MRI scanners.

MRI Scan Type (The Main Field of Scanner)	Exposure Metrics Evaluated over All Samples in the Covered REEs; (R)						
	T, s	SEmf [B, mT]			TVEmf [dB/dt, mT/s]		
	Aver	Aver	95th	Max	Aver	95th	Max
Cardiac MRI	----	----	----	----	----	----	----
CMR-MF (1.5T) [N = 12]	190 (150–240)	57 (40–75)	270 (170–430)	750	44 (30–**58**)	200 (140–270)	1300
CMR-HF (3T) [N = 16]	204 (170–240)	70 (45–110)	390 (220–570)	1300	53 (34–**72)**	250 (170–330)	**2300**
CMR-UHF (7T) [N = 14]	175 (150–220)	70 (33–85)	330 (110–420)	1500	56 (30–**82**)	230 (120–330)	**2900**
CMR-All [N = 42]	190 (150–240)	66 (33–110)	330 (110–570)	1500	51 (30–**82**)	230 (120–330)	**2900**
Head MRI	----	----	----	----	----	----	----
HMR-MF (1.5T) [N = 13]	170 (72–410)	29 (6–65)	98 (17–250)	310	17 (11–35)	78 (14–150)	410
Notes: N—number of REEs in particular set of recordings; T—the duration of the REE; Aver—arithmetic mean value; 95th—the 95th percentile value; Max—maximum value; REE—radiographer exposure event; R—the range between the minimum and maximum values of a particular parameter evaluated over each REE in the set of considered recordings. The evaluation of the probability of vertigo perception: medium vertigo probability (MVP) is marked by underlined values, and high vertigo probability (HVP) is shown in bold. Averaged values were compared with the criteria regarding the parameters used in the study by Schaap [25], which were time averaged over exposure inside a scan room (TA…@r), and 95th percentiles and the Max values were compared with the parameters considered as peak (actual) values during a shift (P…@s).							
